# Magnetic resonance imaging findings in diseases affecting the cranial nerves

**DOI:** 10.1590/0100-3984.2022.55.1e1

**Published:** 2022

**Authors:** Bruno Niemeyer de Freitas Ribeiro

**Affiliations:** Instituto Estadual do Cérebro Paulo Niemeyer, Rio de Janeiro, RJ, Brazil. Email: bruno.niemeyer@hotmail.com.

The 12 cranial nerves represent functional extensions of the brain, connecting the central nervous system with the head and neck, as well as with the thorax and abdomen. Numerous diseases can cause cranial nerve dysfunction, and the assessment of such diseases represents a major diagnostic challenge. Magnetic resonance imaging (MRI) is the main imaging method used in order to evaluate lesions of the central nervous system, and such evaluations have been the subject of a series of recent studies in the radiology literature of Brazil(^1^, ^2^, ^3^, ^4^, ^5^). The use of MRI is essential for the assessment of cranial nerve injuries, and knowledge of MRI acquisition protocols and techniques, as well as of the MRI aspects of the various diseases, is essential for radiologists.

The article authored by Dalaqua et al.^(6)^, published in this issue of **Radiologia Brasileira,** clearly illustrates the main infectious, neoplastic, inflammatory, and demyelinating diseases that can affect the cranial nerves.

When the infectious diseases that can affect the cranial nerves are considered, it should be borne in mind that there has been a recent increase in the incidence of syphilis, and that the first sign of neurosyphilis can be involvement of one or more cranial nerves, in some cases mimicking other, more common, diseases such as schwannoma(^7^,^8^). Within the context of the epidemiology of Brazil, meningeal tuberculosis and leptospirosis also continue to be of concern, the latter being quite strongly associated with poor sanitation and flooding, especially during the summer(^9^,^10^).

Among the neoplastic diseases that can affect the cranial nerves, the most common intracranial tumor in adults is meningioma, which is benign in the vast majority of cases. In the evaluation of schwannomas, which are commonly found in the eighth cranial nerve, MRI can serve not only to monitor the lesion but also to guide the choice of the most appropriate surgical approach, as well as to determine the prognosis, including the likelihood that the individual will recover their hearing^(11)^. However, radiologists should also be familiar with malignant neoplastic causes of cranial nerve involvement, mainly because of the aging of the population and the consequent increase in the number of cases of cancer, especially leptomeningeal carcinomatosis with cranial nerve involvement (which is most common in cases of breast and lung cancer) and lymphoma.

Among demyelinating diseases and other diseases that affect the cranial nerves, the most common disabling disease of the central nervous system in young adults is multiple sclerosis. In patients with multiple sclerosis, involvement of cranial nerve nuclei can provoke symptoms that often mimic those of trigeminal neuralgia. It is therefore necessary to exclude multiple sclerosis in young patients, especially female patients, with symptoms suggestive of trigeminal neuralgia. In the context of demyelinating diseases and other diseases involving the cranial nerves, clinical and biochemical findings, as well as imaging findings (including those from modalities other than MRI), are quite useful in making a definitive diagnosis, such findings including symmetrical proximal and distal weakness with sensory loss, in chronic inflammatory demyelinating polyneuropathy; positivity for anti-aquaporin 4 antibodies, in neuromyelitis optica spectrum disorders; and typical chest computed tomography findings, in sarcoidosis.

During the coronavirus disease 2019 pandemic, numerous neurological manifestations, including involvement of the seventh cranial nerve, were described as complications of the disease(^12^,^13^). Such manifestations were most often attributed to an immune-mediated injury rather than to direct viral neurotropism.

In conclusion, as suggested by Dalaqua et al.^(6)^, radiologists should be prepared to interpret neuroimaging findings in cases of cranial nerve involvement. Despite not being pathognomonic, such findings help narrow the differential diagnosis. Taken together with clinical and laboratory data, neuroimaging findings can also guide the final diagnosis and inform decisions regarding the therapeutic approach.
